# Supplementary material for: Physics-based models outperform AI weather forecasts of record-breaking extremes
Source: Sci Adv. 2026 Apr 29;12(18):eaec1433. doi: 10.1126/sciadv.aec1433 (PMC13127558; doi:10.1126/sciadv.aec1433)
Supplement: Supplementary file 1 — Figs. S1 to S21 [file sciadv.aec1433_sm.pdf]

Science Advances
AAAS

Supplementary Materials for

Physics-based models outperform AI weather forecasts of record-breaking extremes

Zhongwei Zhang *et al.*

Corresponding author: Zhongwei Zhang, zhongwei.zhang@kit.edu;
Sebastian Engelke, sebastian.engelke@unige.ch

Sci. Adv. **12**, eaec1433 (2026)
DOI: 10.1126/sciadv.aec1433

This PDF file includes:

Figs. S1 to S21

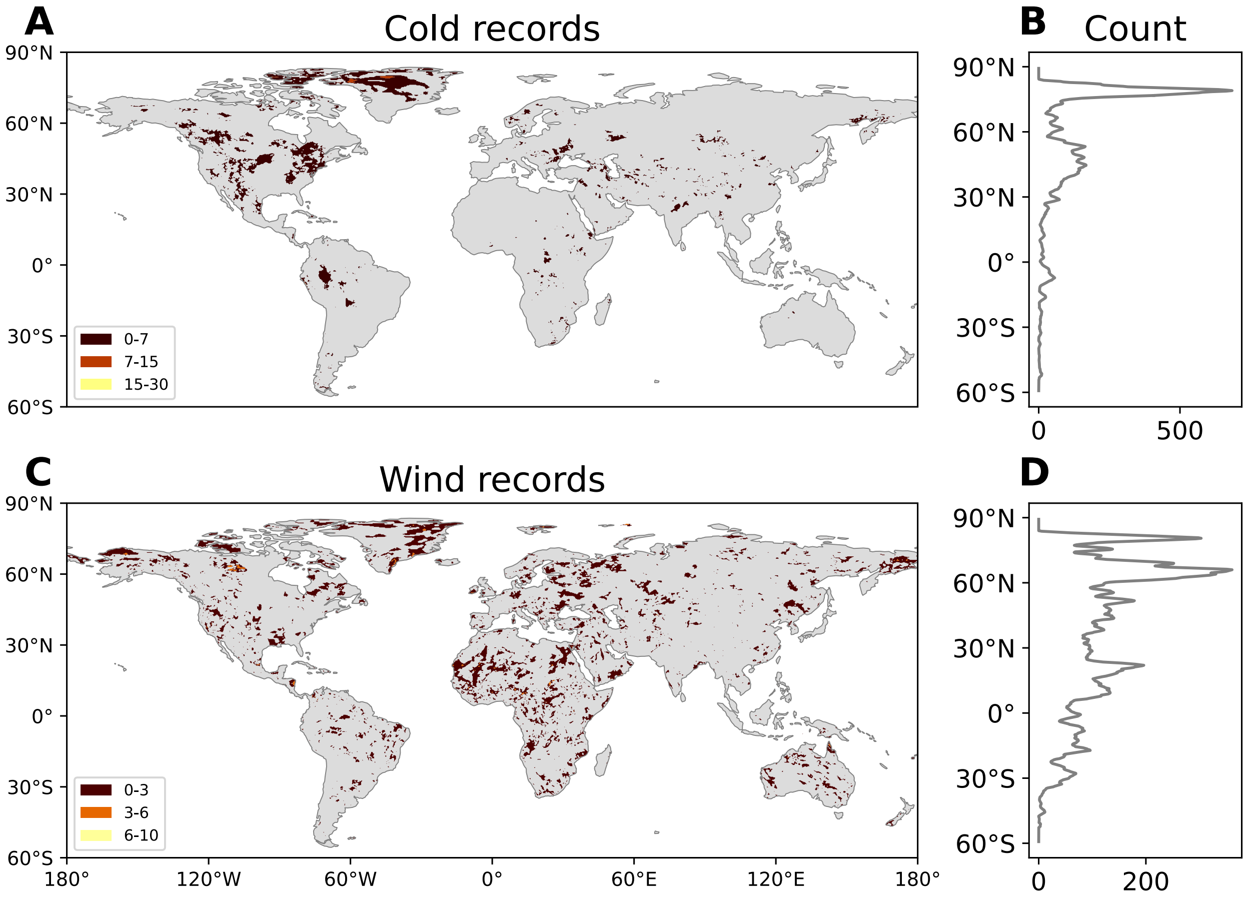

Figure S1: **Number of record-breaking events over land (excluding the Antarctic region) in 2020 in ERA5**. (**A** and **C**) Number of cold and wind records. (**B** and **D**) Number of cold and wind records per latitude.

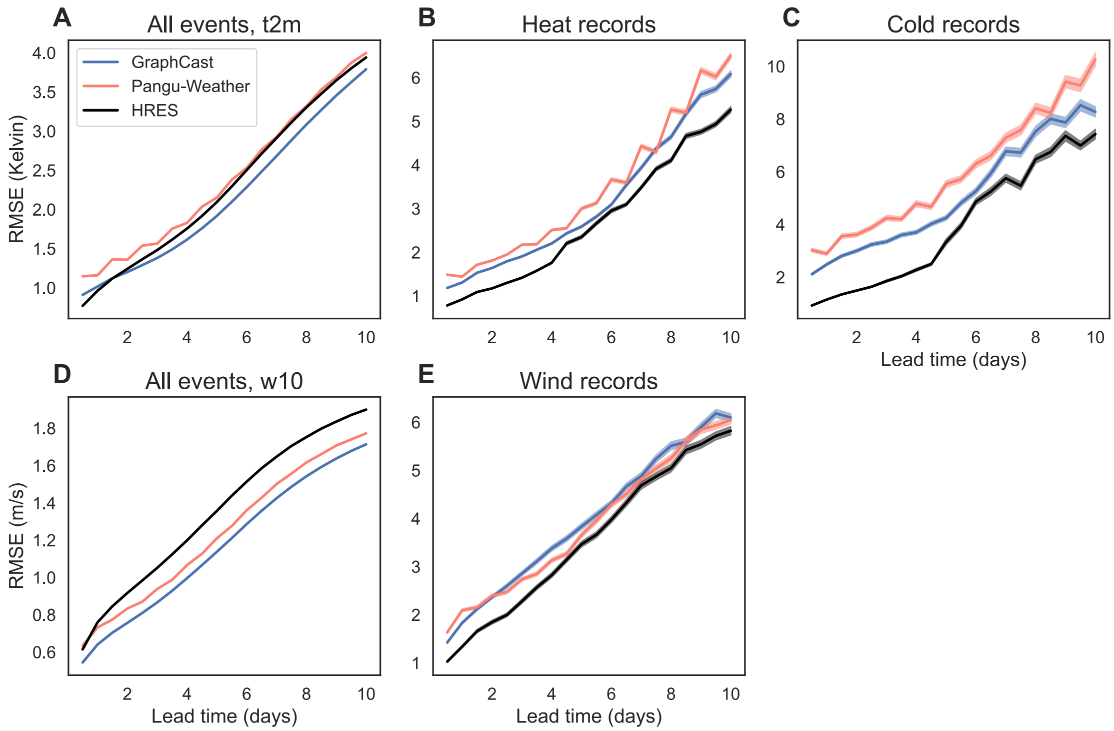

Figure S2: **Model performance on all events and record-breaking events in 2018**. (**A–E**) RMSE of 2m temperature and 10m wind speed over land (excluding the Antarctic region) of HRES, Pangu-Weather, and GraphCast for all events (**A**, **D**) and only record-breaking events (**B**, **C**, **E**) in 2018. The transparent shaded areas indicate 95% confidence bands.

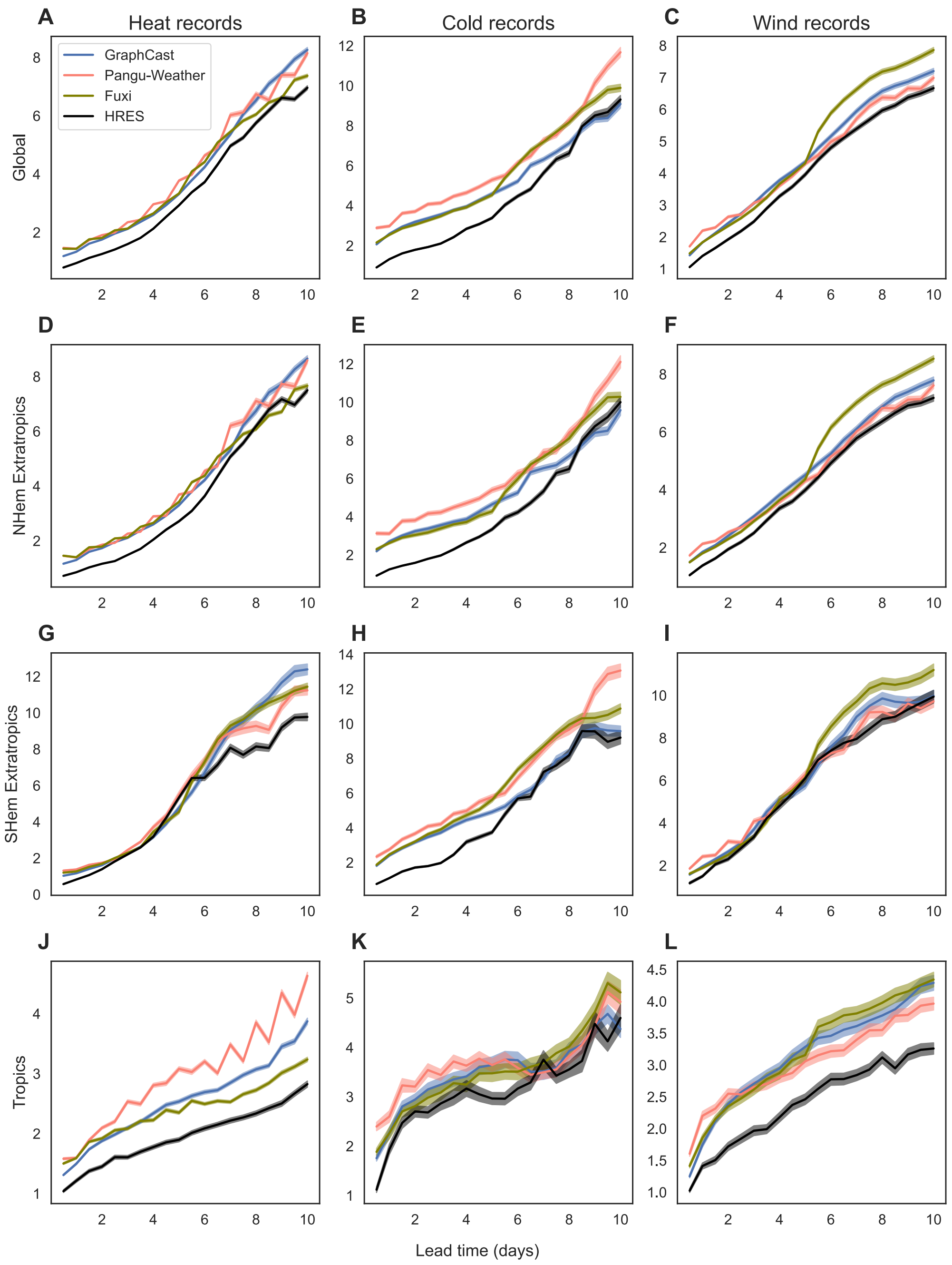

Figure S3: **Regional RMSE of 2m temperature and 10m wind speed over land in 2020**. (**A–C**) RMSE for the whole globe. (**D–F**) RMSE for Northern Hemisphere Extratropics (NHem Extratropics, i.e., grid cells with latitude in $[20, 90]$). (**G–I**) RMSE for Southern Hemisphere Extratropics (SHem Extratropics, i.e., grid cells with latitude in $[-90, -20]$). (**J–L**) RMSE for Tropics (grid cells with latitude in $[-20, 20]$). The transparent shaded areas indicate 95% confidence bands.

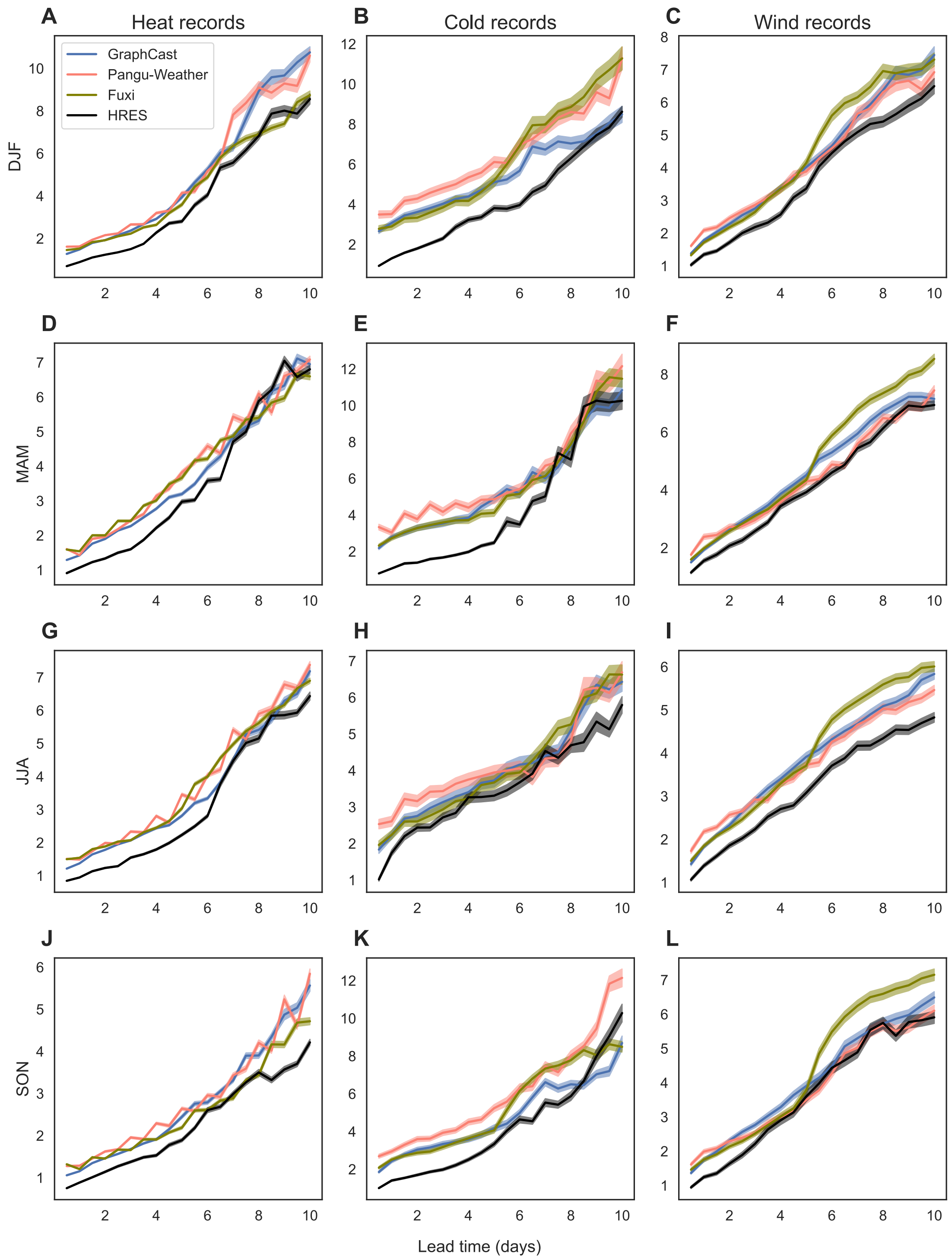

Figure S4: **Seasonal RMSE of 2m temperature and 10m wind speed over land (excluding the Antarctic region) in 2020**. (**A–C**) RMSE for the months December – February. (**D–F**) RMSE for the months March – May. (**G–I**) RMSE for the months June – August. (**J–L**) RMSE for the months September – November.

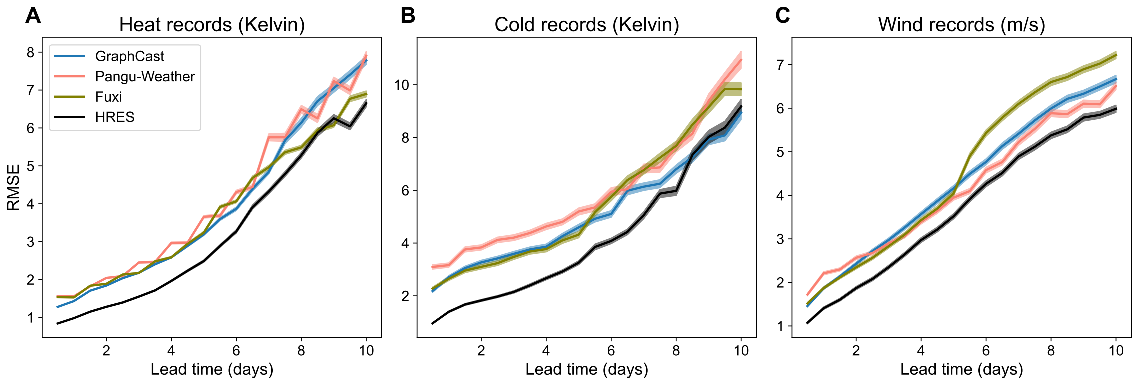

Figure S5: **Model performance on record-breaking events which have the largest record exceedance per month per grid point.** (**A–C**) Root mean square error (RMSE) of forecasted 2m temperature and 10m wind speed over land (excluding the Antarctic region) of HRES, Pangu-Weather, GraphCast, and Fuxi for record-breaking events with the largest exceedances per month per grid point at 00/12 UTC in 2020 for different lead times. The transparent shaded areas indicate 95% confidence bands.

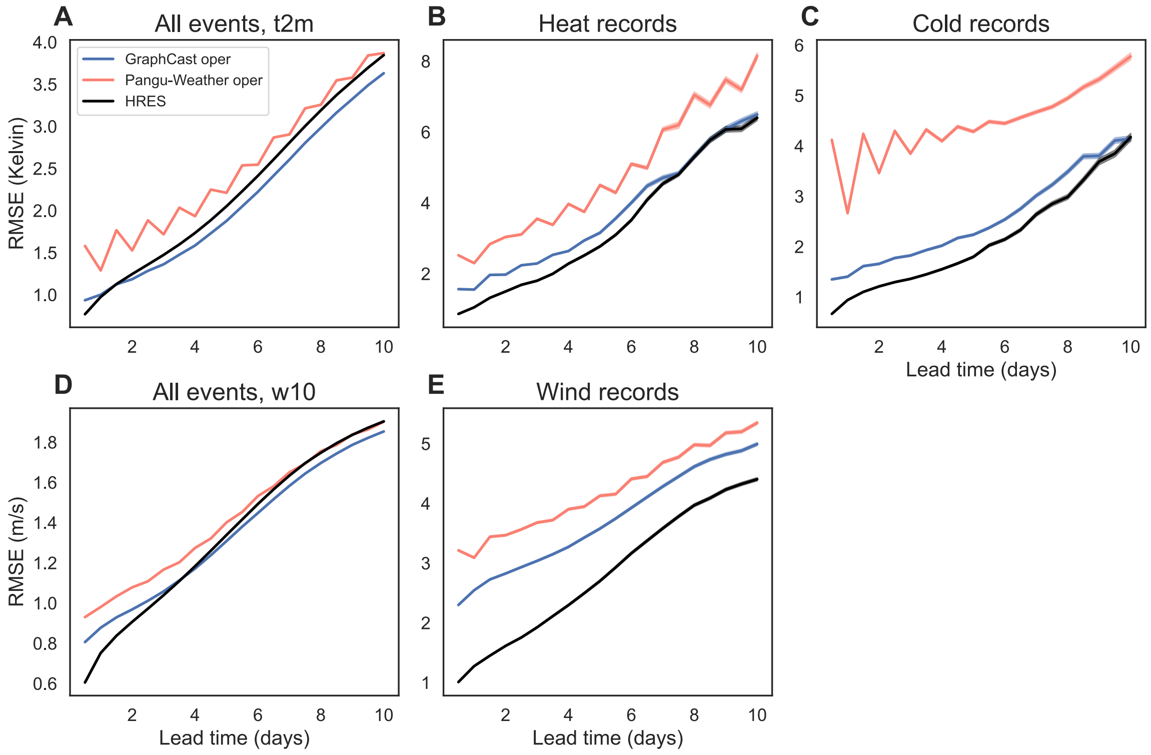

Figure S6: **Model performance of operational forecasts on all events and record-breaking events in 2020.** RMSE of 2m temperature and 10m wind speed over land (excluding the Antarctic region) of HRES, Pangu-Weather operational, and GraphCast operational for all events (**A**, **D**) and only record-breaking events (**B**, **C**, **E**) in 2020. The transparent shaded areas indicate 95% confidence bands.

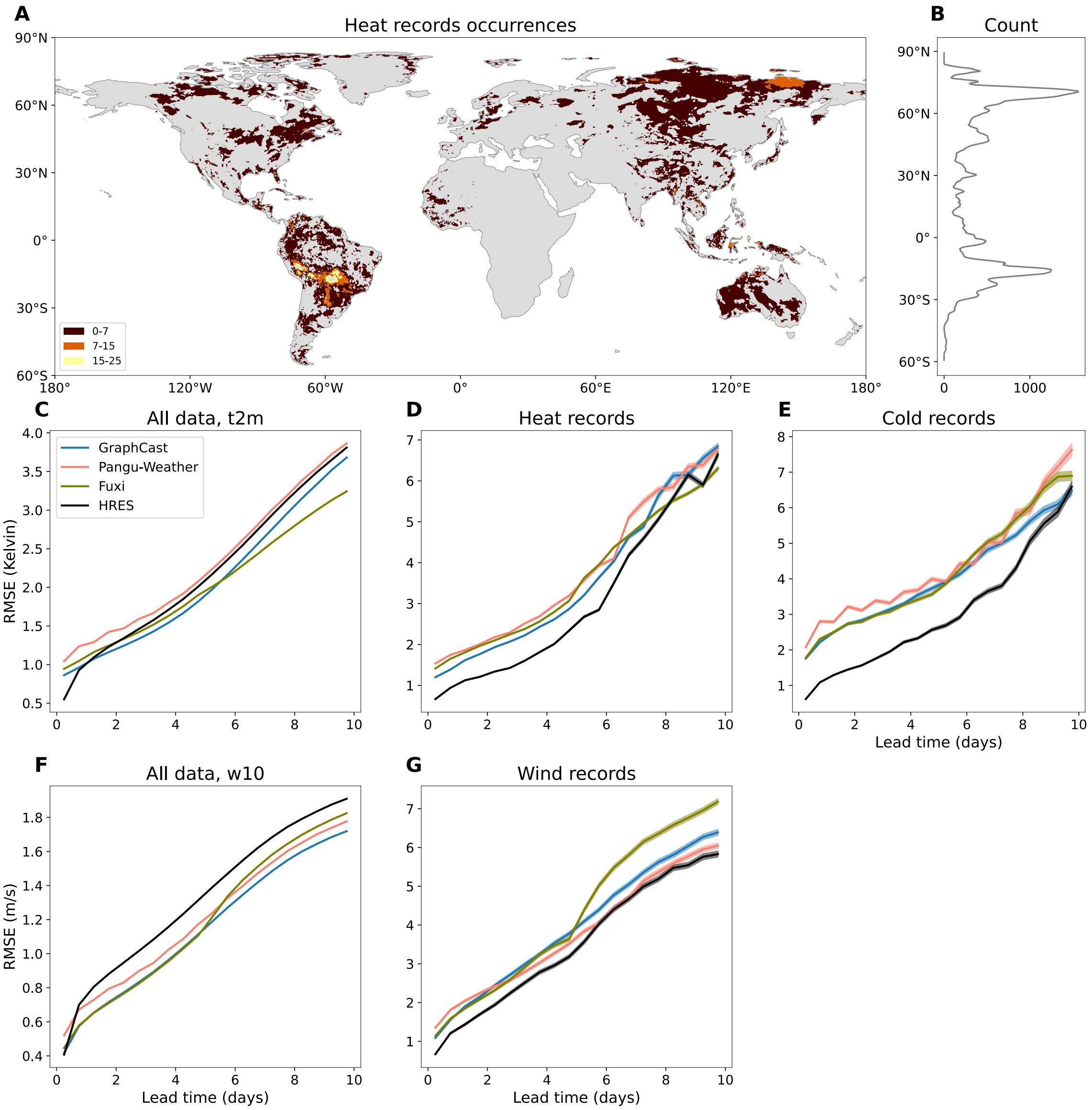

Figure S7: **Model performance on all events and record-breaking events at 06/18 UTC.** (**A**) Number of heat records at 06/18 UTC in 2020 in ERA5. (**B**) Number of heat records per latitude. (**C–G**) Root mean square error (RMSE) of forecasted 2m temperature and 10m wind speed over land (excluding the Antarctic region) of HRES, Pangu-Weather, GraphCast, and Fuxi for all data at 06/18 UTC (**C, F**) and only record-breaking events at 06/18 UTC (**D, E, G**) in 2020 for different lead times. The transparent shaded areas indicate 95% confidence bands.

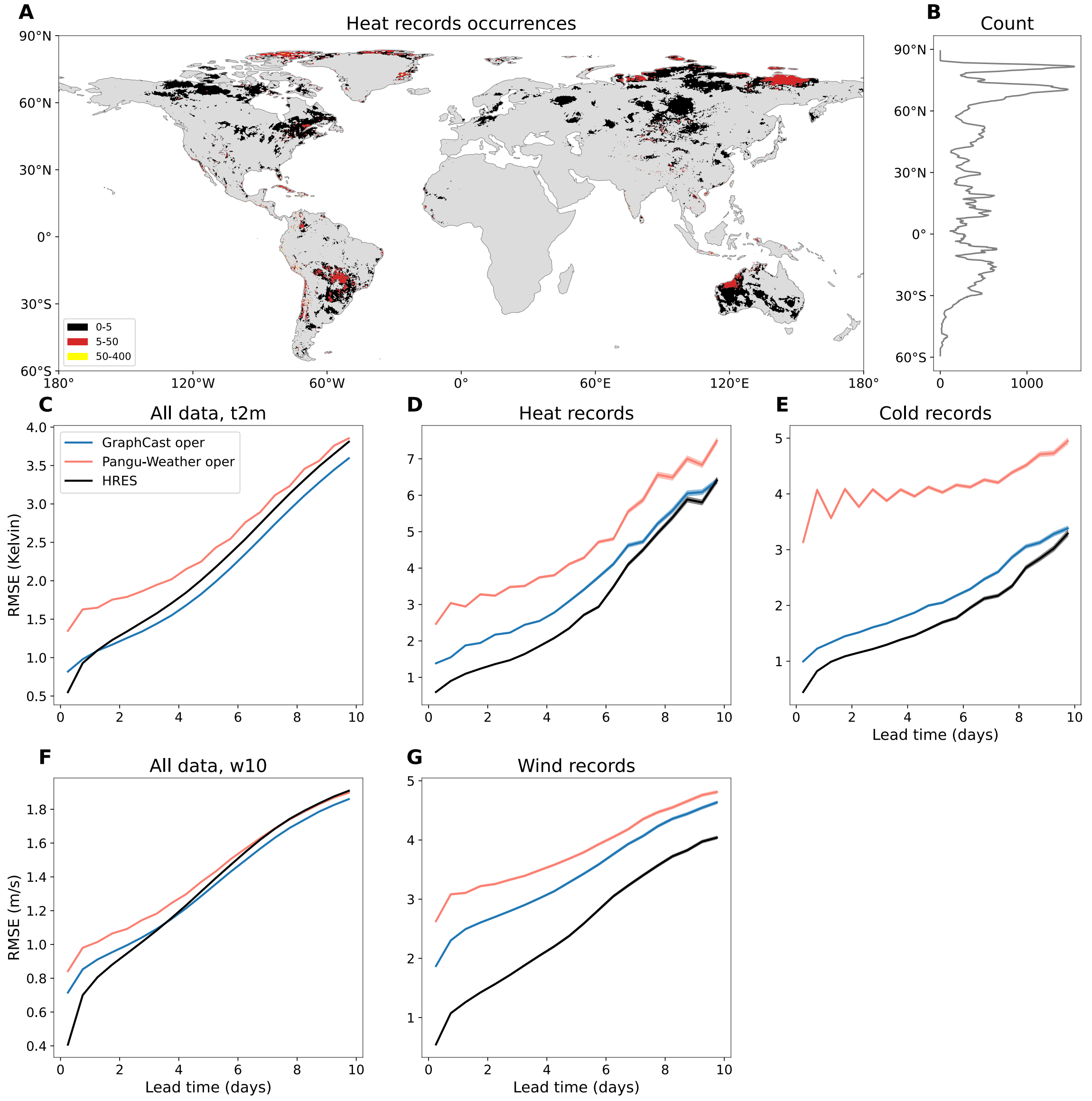

Figure S8: **Performance of operational models on all events and record-breaking events at 06/18 UTC.** (**A**) Number of heat records at 06/18 UTC in 2020 in HRES-fc0. (**B**) Number of heat records per latitude. (**C–G**) Root mean square error (RMSE) of forecasted 2m temperature and 10m wind speed over land (excluding the Antarctic region) of HRES, Pangu-Weather operational, and GraphCast operational for all data at 06/18 UTC (**C, F**) and only record-breaking events at 06/18 UTC (**D, E, G**) in 2020 for different lead times. The transparent shaded areas indicate 95% confidence bands.

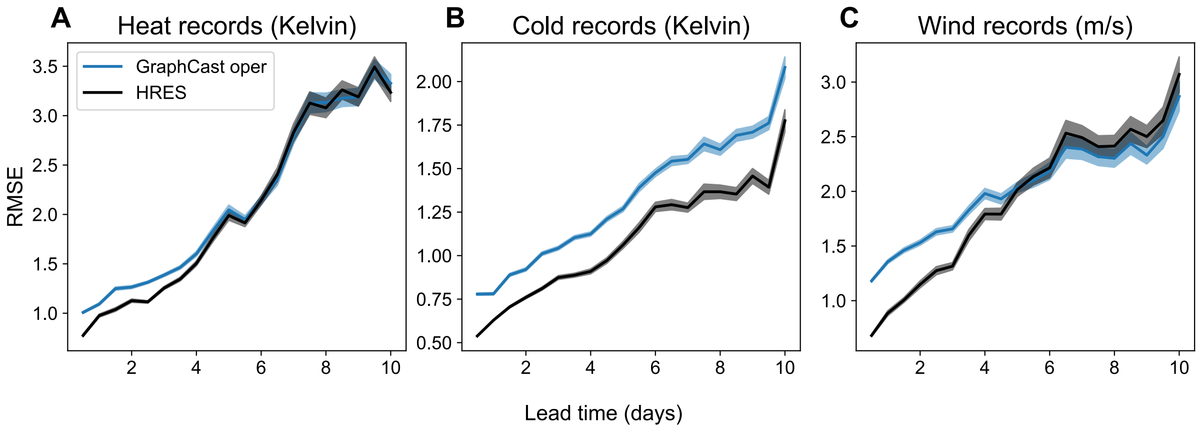

Figure S9: **RMSE when conditioning on the forecasts rather than the observational ground truth.** RMSE of heat (**A**), cold (**B**), and wind records (**C**) over land (excluding the Antarctic region) in 2020, when the record-breaking events are selected using the HRES and GraphCast operational forecasts (when both of them issue a record-breaking forecast). The transparent shaded areas indicate 95% confidence bands.

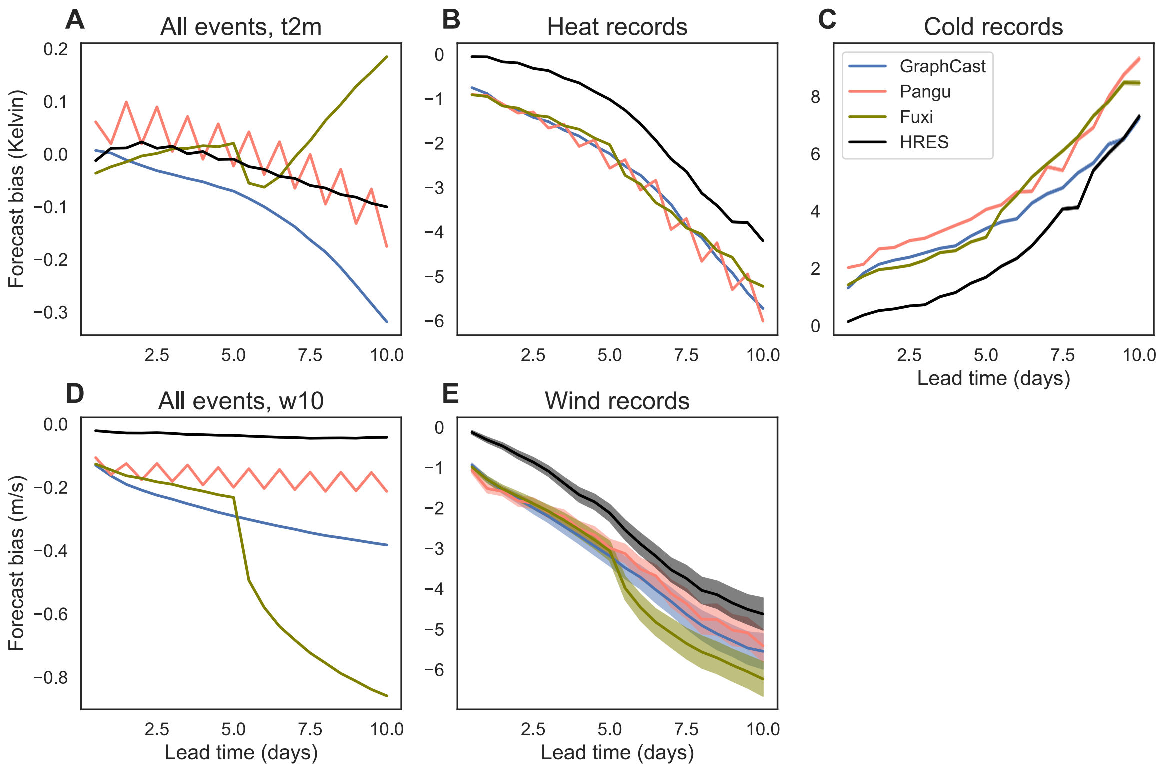

Figure S10: **Forecast bias for all and record-breaking events in 2020**. Forecast bias of 2m temperature and 10m wind speed over land (excluding the Antarctic region) of HRES, GraphCast, Pangu-Weather, and Fuxi for all events (**A**, **D**) and only record-breaking events (**B**, **C**, **E**) in 2020. The transparent shaded areas indicate 95% confidence bands. Pangu-Weather uses two different models (6h and 24h) for different lead times, resulting in the zigzag pattern of its bias.

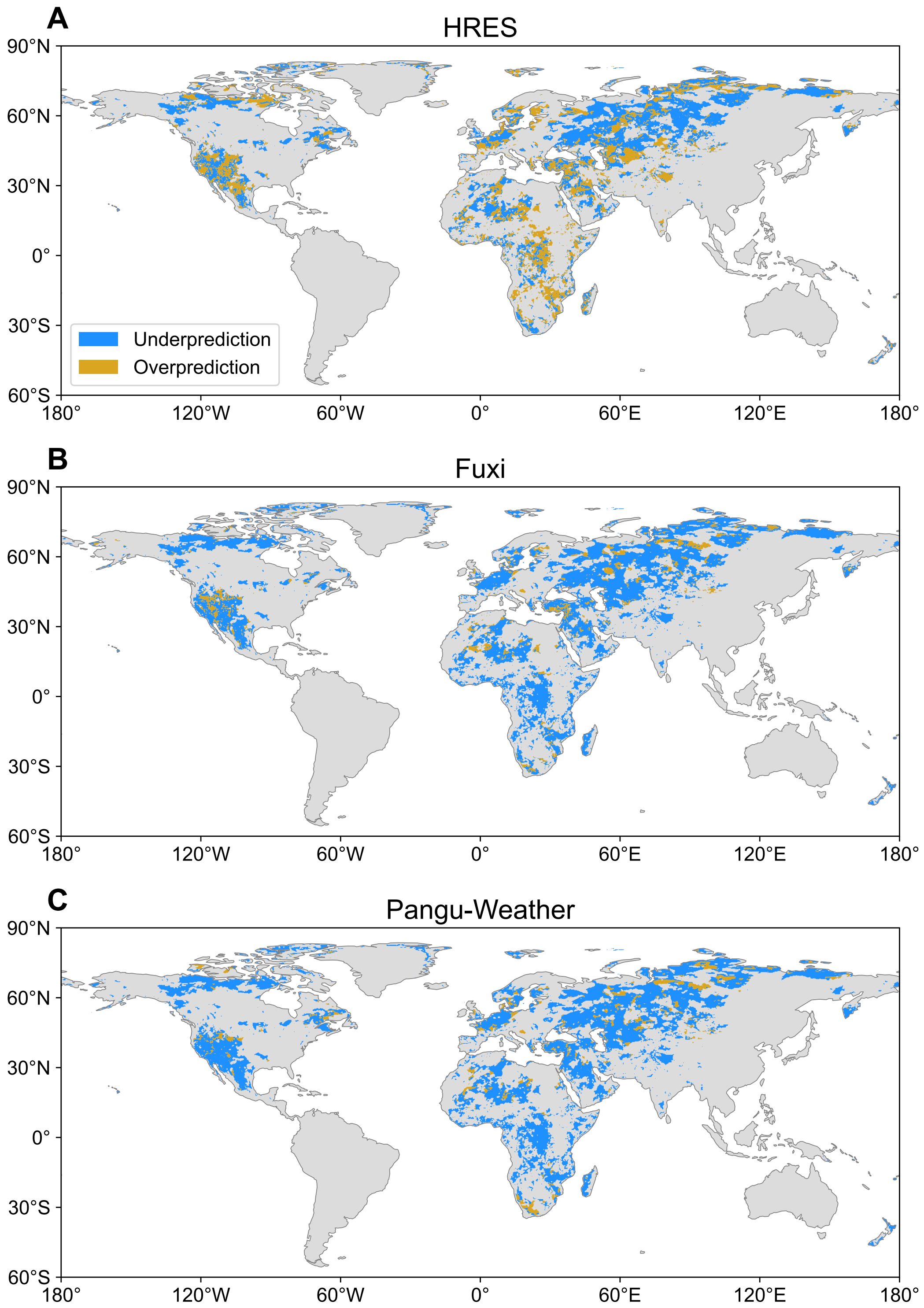

Figure S11: **Forecast bias of lead time 2 days of the maximum heat records for different models.**. (**A**) Forecast bias for the physics-based numerical model HRES. (**B**) Forecast bias for AI model Fuxi. (**C**) Forecast bias for AI model Pangu-Weather.

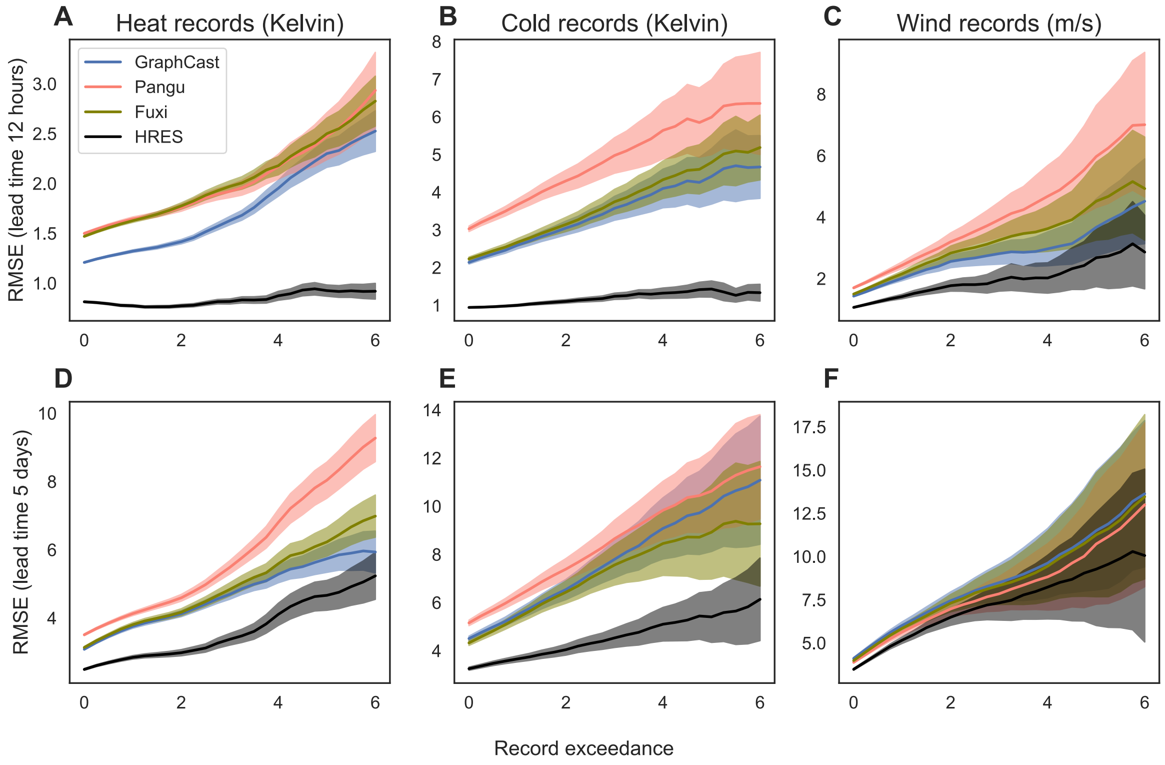

Figure S12: **RMSE against record exceedance for different lead times in 2020.** RMSE of 2m temperature and 10m wind speed for lead time 12 hours (**A–C**) and 5 days (**D–F**) over land (excluding the Antarctic region) in 2020, for events that exceed the record by at least a certain margin (in Kelvin or m/s). The transparent shaded areas indicate 95% confidence bands.

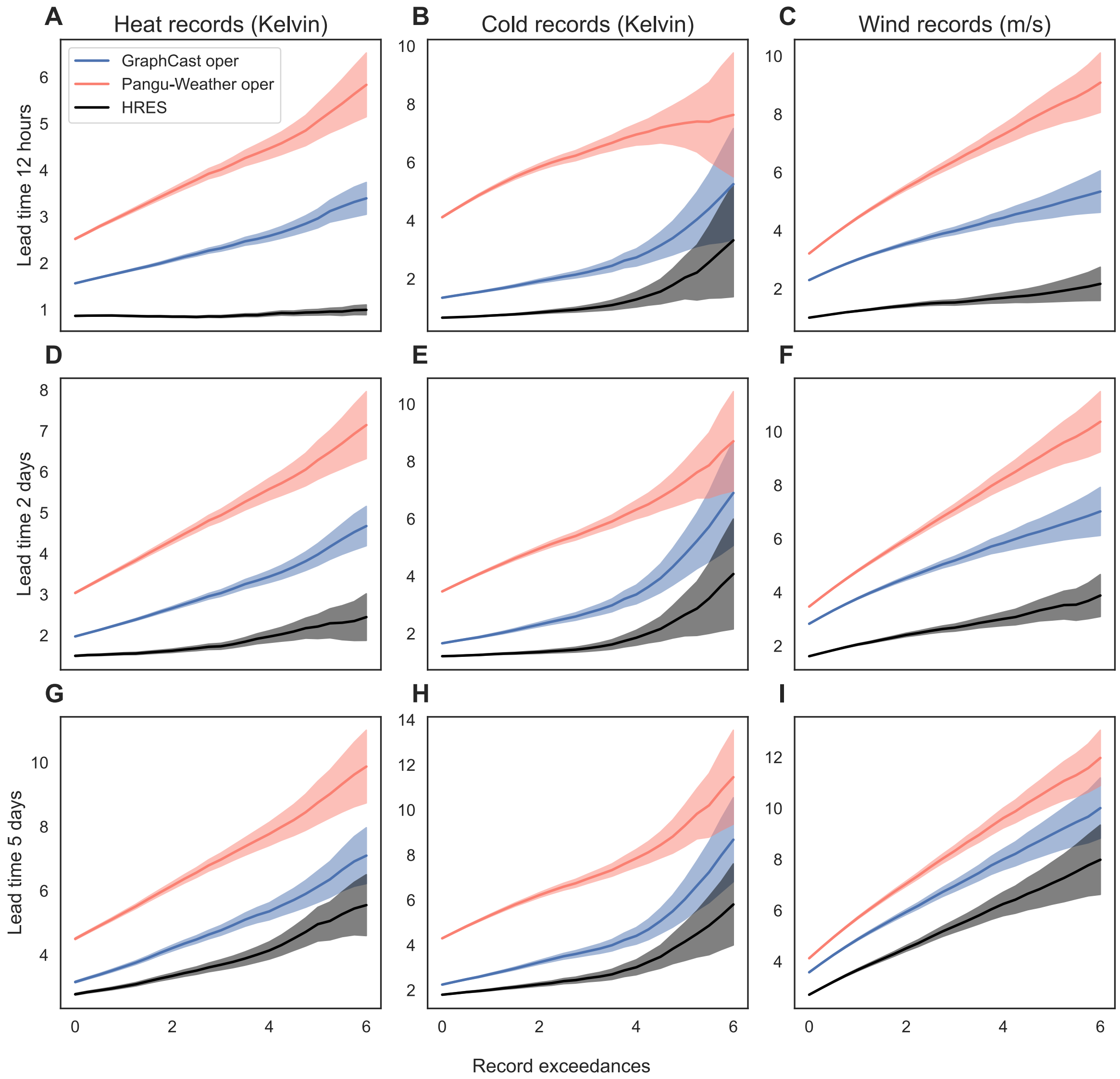

Figure S13: **RMSE of operational forecasts against record exceedances for different lead times in 2020**. RMSE of 2m temperature and 10m wind speed over land (excluding the Antarctic region) of HRES, Pangu-Weather operational, and GraphCast operational in 2020, for events that exceed the record at least by a certain margin, for lead time 12 hours (**A–C**), 2 days (**D–F**), 5 days (**G–I**). The transparent shaded areas indicate 95% confidence bands.

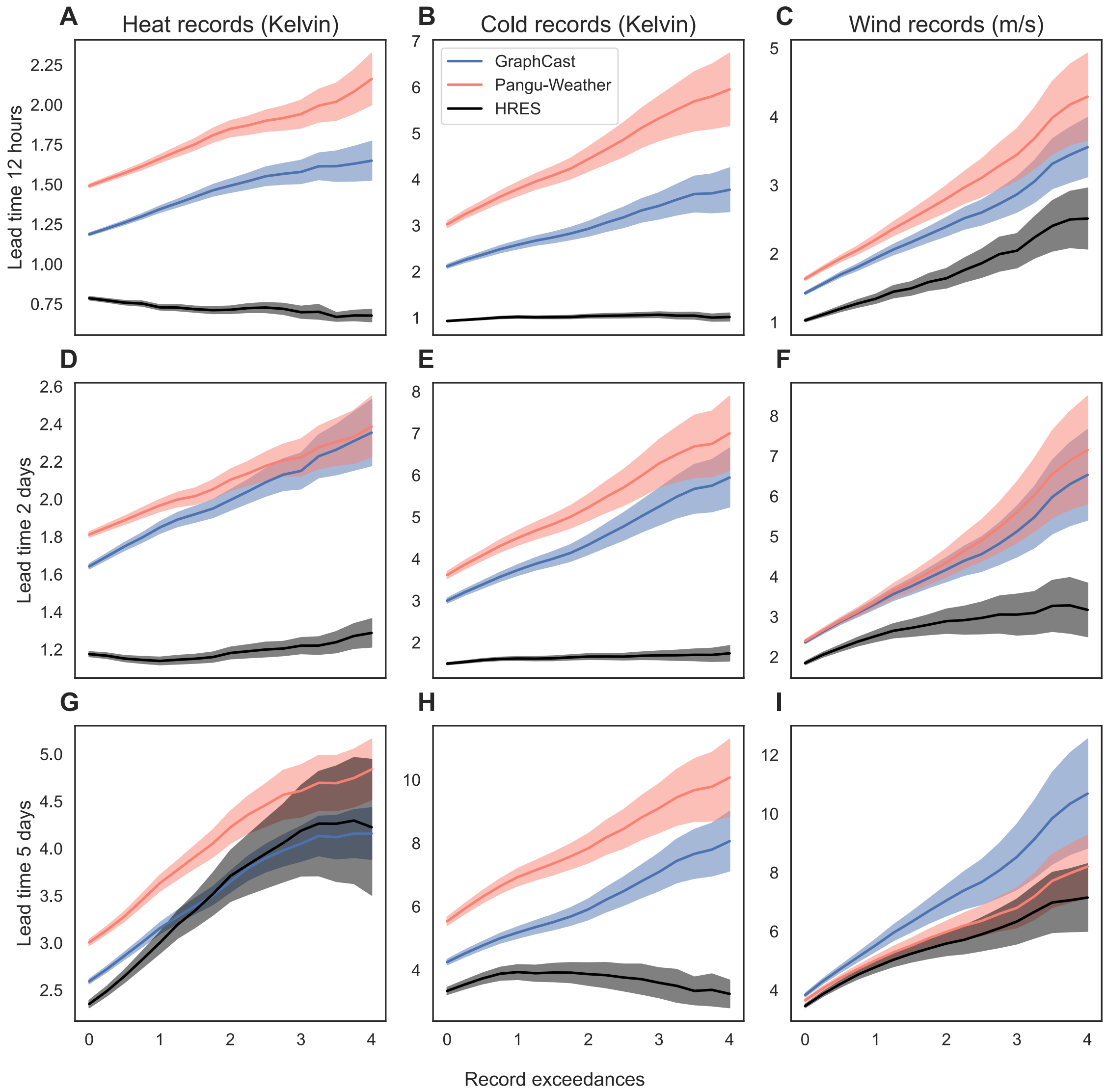

Figure S14: **RMSE against record exceedances in 2018**. RMSE of 2m temperature and 10m wind speed over land (excluding the Antarctic region) of HRES, Pangu-Weather, and GraphCast in 2018, for events that exceed the record by at least a certain margin, for lead time 12 hours (**A–C**), 2 days (**D–F**), and 5 days (**G–I**). The transparent shaded areas indicate 95% confidence bands.

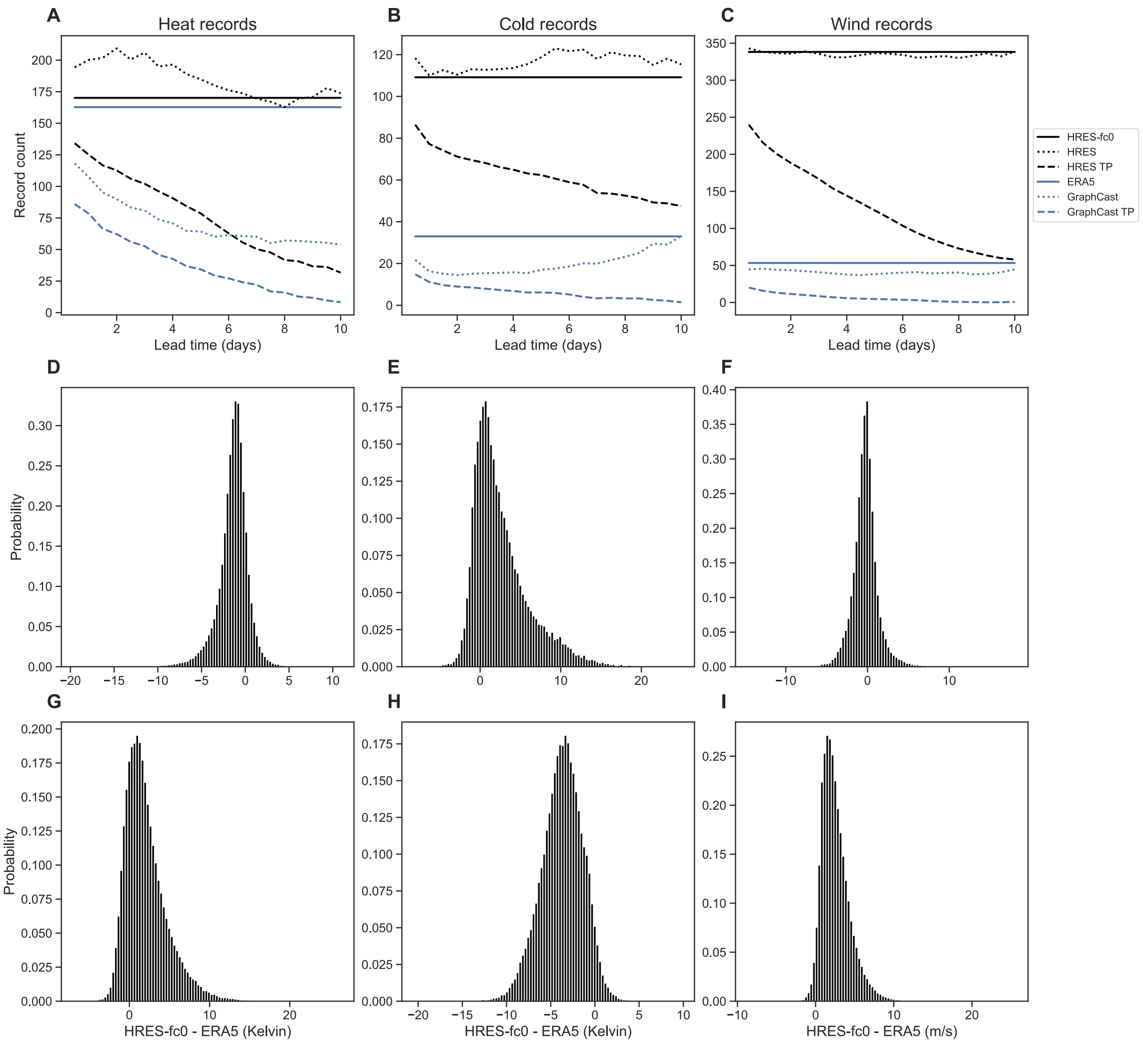

Figure S15: **Further comparison of GraphCast and HRES forecasts of occurrence of record-breaking events over land (excluding the Antarctic region) in 2020**. (**A**-**C**) Counts (in thousands) of heat, cold, and wind records in the ground truth ERA5 and HRES-fc0, and GraphCast and HRES forecast data, as well as counts of their true positives (TP). (**D**-**F**) Histogram of the intensity difference between the two ground truth data (i.e., HRES-fc0 - ERA5) of the record-breaking events identified by ERA5. (**G**-**I**) Histogram of the intensity difference between the two ground truth data (HRES-fc0 - ERA5) of the record-breaking events identified by HRES-fc0. Note that for record-breaking events in ERA5, the HRES-fc0 data at the corresponding time points and grid cells might not necessarily exceed the records. Similarly for record-breaking events in HRES-fc0, ERA5 might not exceed the records either.

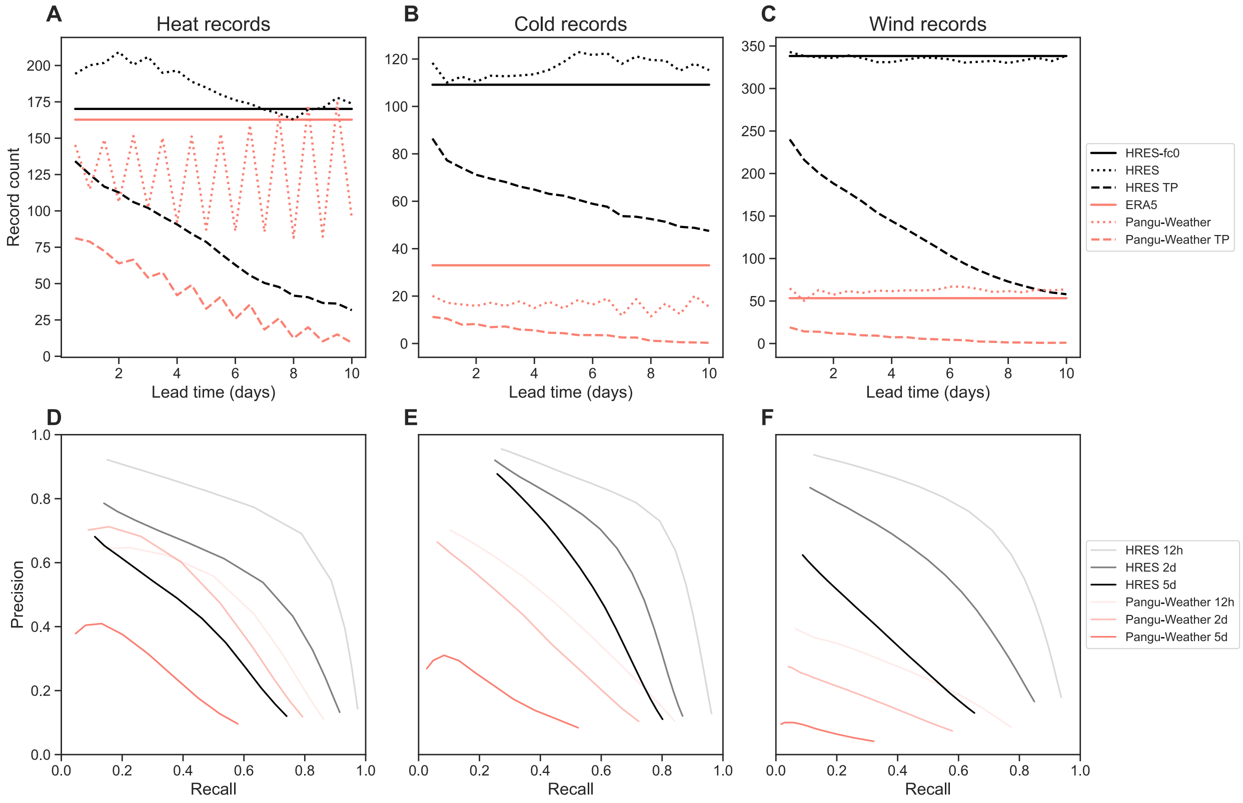

Figure S16: **Pangu-Weather forecast of occurrence of record-breaking events over land (excluding the Antarctic region) in 2020**. (**A-C**) Counts (in thousands) of heat, cold, and wind records in the ground truth ERA5 and HRES-fc0, and Pangu-Weather and HRES forecast data, as well as counts of their true positives (TP). (**D-F**) Precision and recall curve of Pangu-Weather and HRES forecasts when using the record data as the threshold.

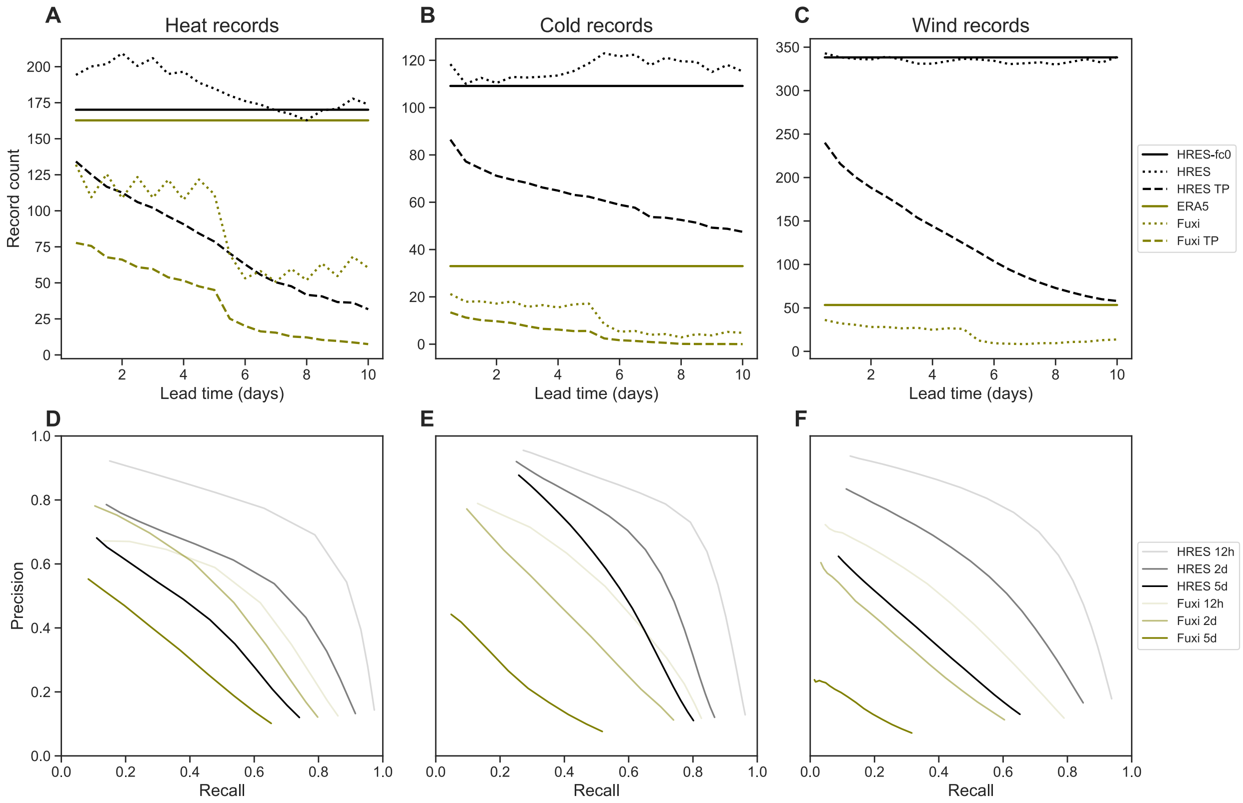

Figure S17: **Fuxi forecast of occurrence of record-breaking events over land (excluding the Antarctic region) in 2020**. (**A-C**) Counts (in thousands) of heat, cold, and wind records in the ground truth ERA5 and HRES-fc0, and Fuxi and HRES forecast data, as well as counts of their true positives (TP). (**D-F**) Precision and recall curve of Fuxi and HRES forecasts when using the record data as the threshold.

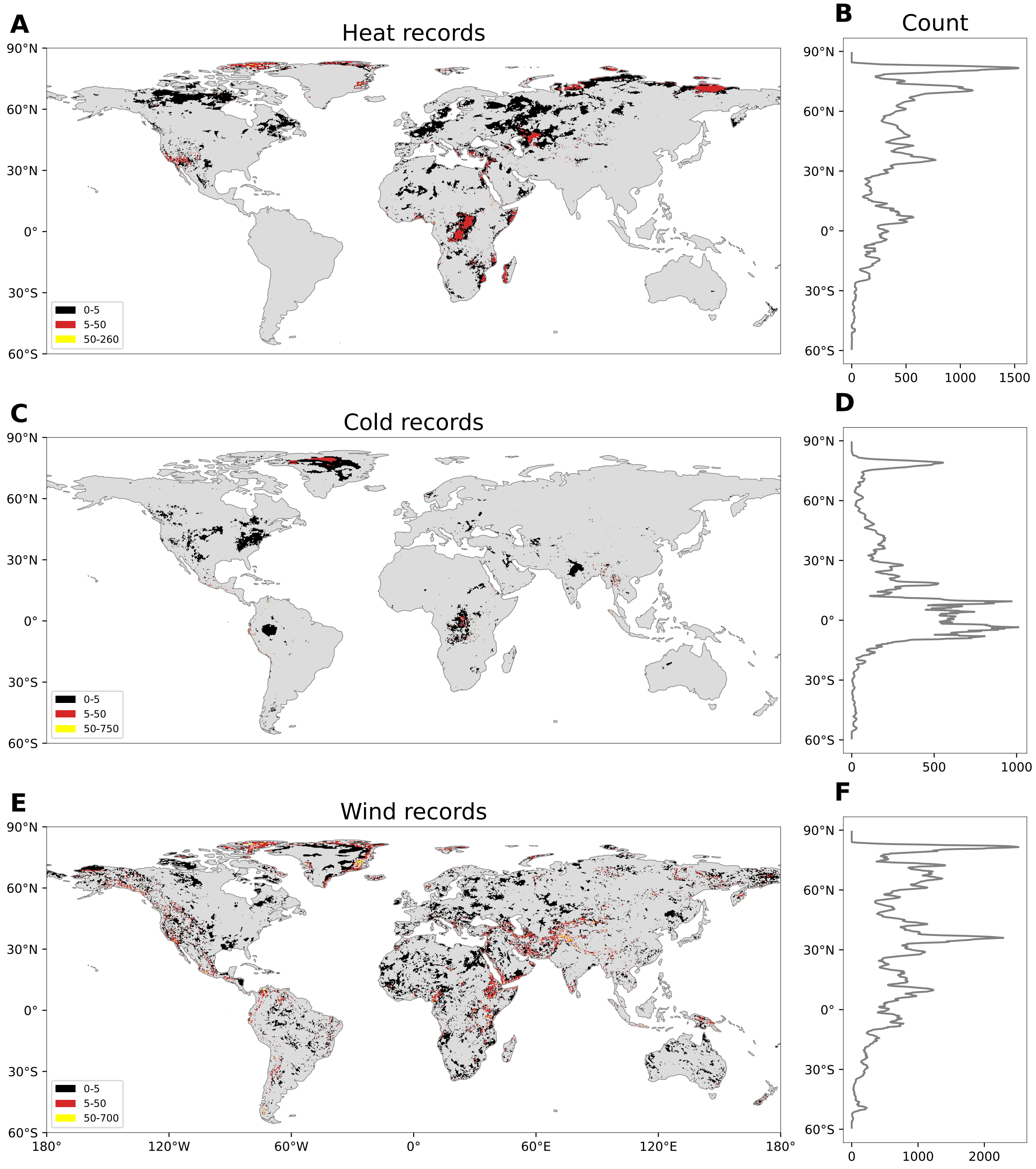

Figure S18: **Number of records over land (excluding the Antarctic region) in 2020 in HRES-fc0**. (**A**, **C**, and **E**) Number of heat, cold, and wind records. (**B**, **D**, and **F**) Number of heat, cold, and wind records per latitude.

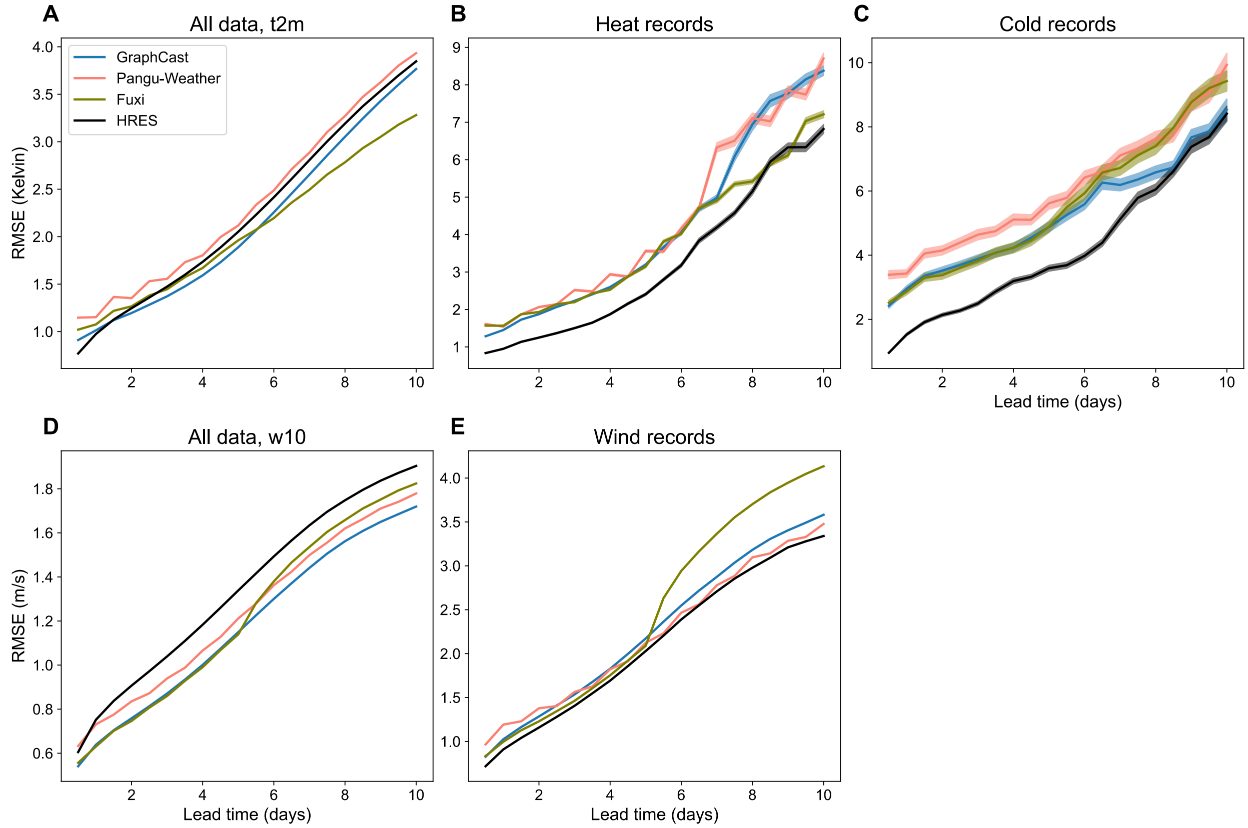

Figure S19: **Model performance on all events and record-breaking events in 2020, when a 31-day running window is used to define the records.** RMSE of 2m temperature and 10m wind speed over land (excluding the Antarctic region) of HRES, Pangu-Weather, GraphCast, and Fuxi for all events (**A**, **D**) and only record-breaking events (**B**, **C**, **E**) in 2020. The transparent shaded areas indicate 95% confidence bands.

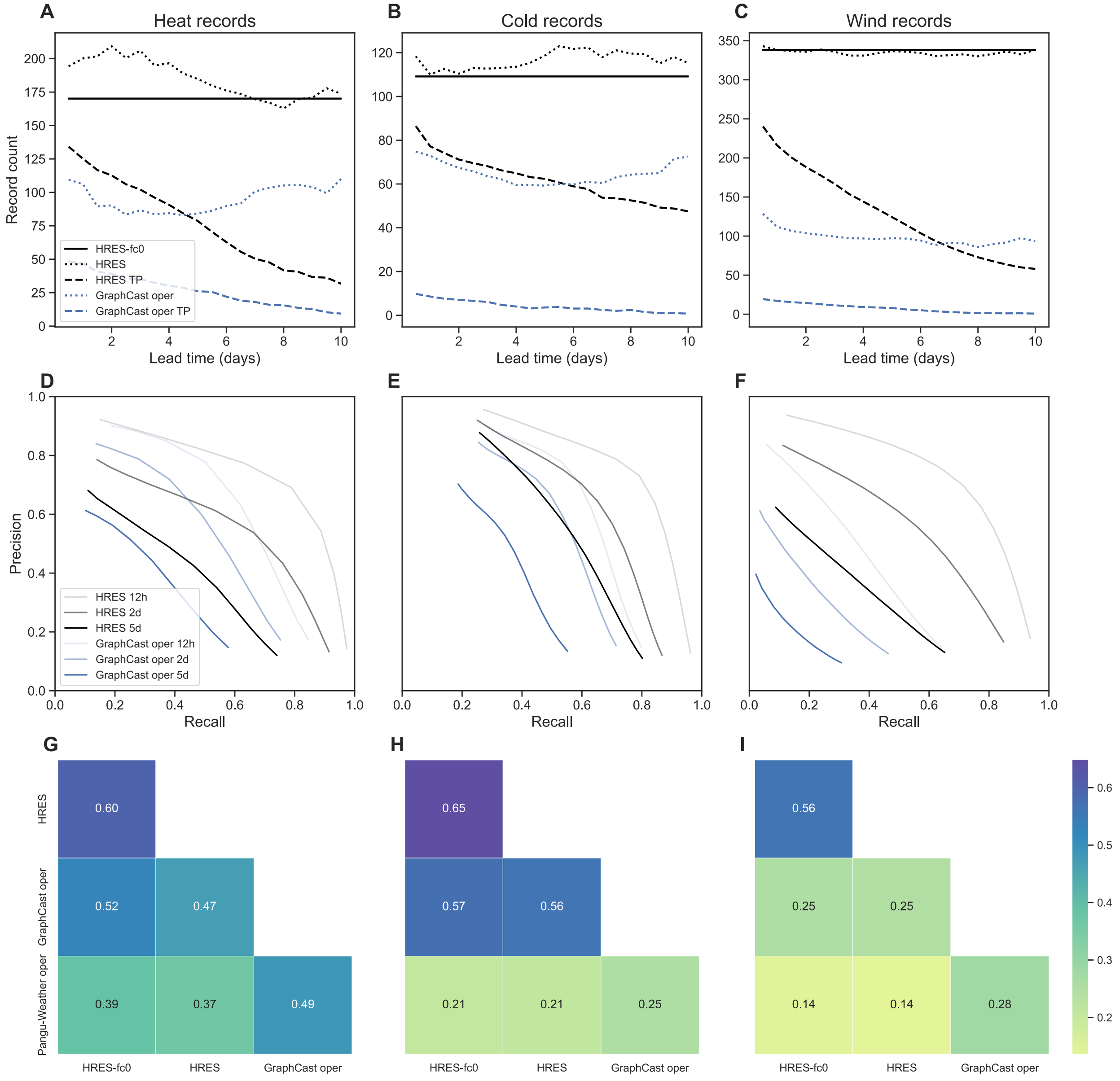

Figure S20: **Operational forecasts of the occurrence of record-breaking events**. (**A–C**) counts (in thousands) of heat, cold, and wind records over land (excluding the Antarctic region) in 2020 in the ground truth HRES-fc0 data, and GraphCast operational and HRES forecast data, as well as counts of their true positives (TP). (**D–F**) Precision and recall curve of GraphCast operational and HRES forecasts when the record data are used as the threshold. (**G–I**) Correlations between the indicator functions of whether the ground truth or 2-day forecast data exceed the record.

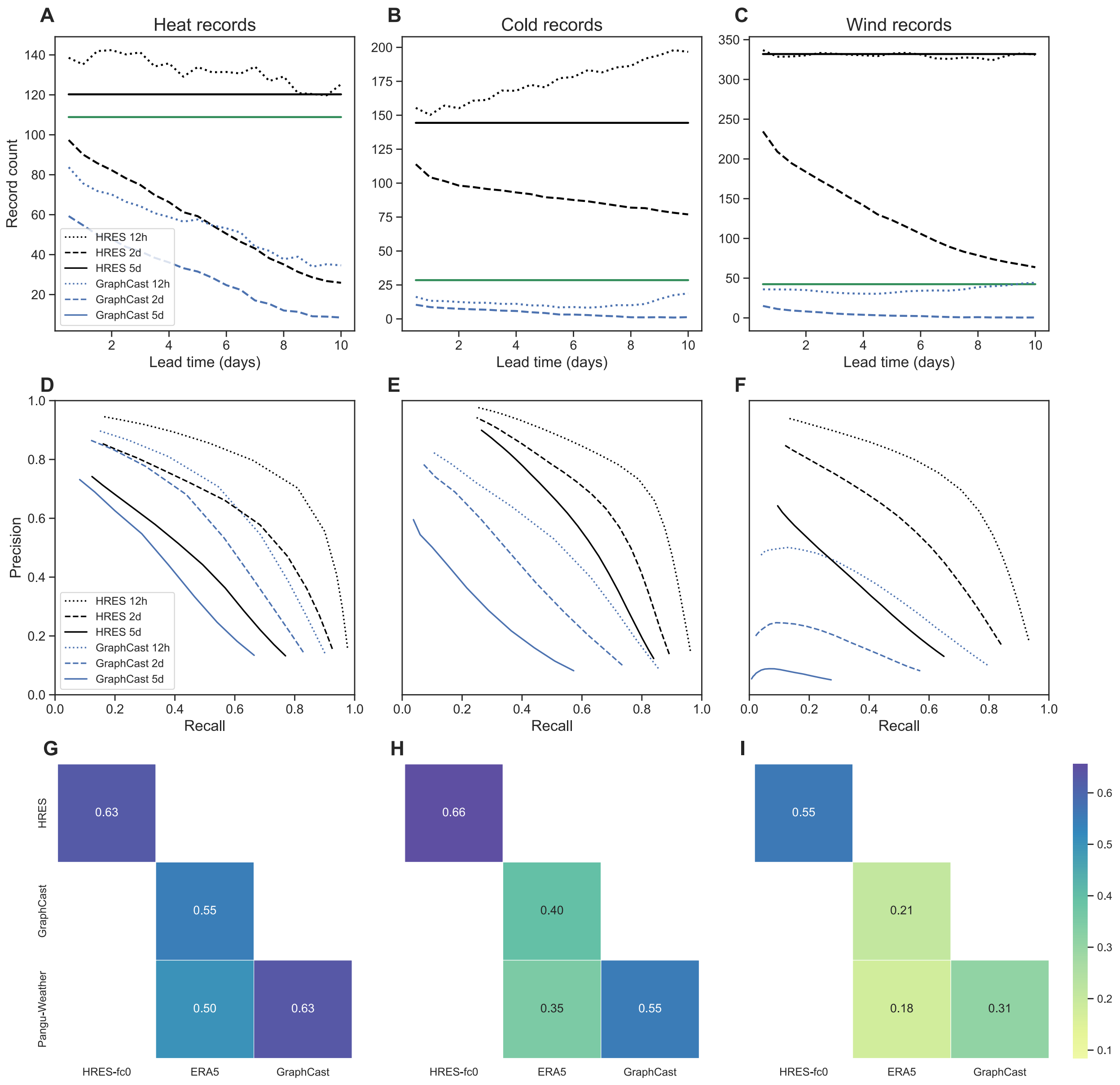

Figure S21: **Forecasts of the occurrence of record-breaking events in 2018**. (**A–C**) counts (in thousands) of heat, cold, and wind records over land (excluding the Antarctic region) in 2018 in the ground truth HRES-fc0 data, and GraphCast and HRES forecast data, as well as counts of their true positives (TP). (**D–F**) Precision and recall curve of GraphCast and HRES forecasts when the record data are used as the threshold. (**G–I**) Correlations between the indicator functions of whether the ground truth or 2-day forecast data exceed the record.
